# A Deconvolutional Deblurring Algorithm Based on Short- and Long-Exposure Images

**DOI:** 10.3390/s22051846

**Published:** 2022-02-25

**Authors:** Yang Bai, Zheng Tan, Qunbo Lv, Min Huang

**Affiliations:** 1 Aerospace Information Research Institute, Chinese Academy of Sciences, No.9 Dengzhuang South Road, Haidian District, Beijing 100094, China; baiyang171@mails.ucas.ac.cn (Y.B.); tanzheng@aircas.ac.cn (Z.T.); lvqunbo@aoe.ac.cn (Q.L.); 2 School of Optoelectronics, University of Chinese of Academy Sciences, No.19(A) Yuquan Road, Shijingshan District, Beijing 100039, China; 3 Department of Key Laboratory of Computational Optical Imagine Technology, CAS, No.9 Dengzhuang South Road, Haidian District, Beijing 100094, China

**Keywords:** short-exposure image, long-exposure image, kernel estimation

## Abstract

An iterative image restoration algorithm, directed at the image deblurring problem and based on the concept of long- and short-exposure deblurring, was proposed under the image deconvolution framework by investigating the imaging principle and existing algorithms, thus realizing the restoration of degraded images. The effective priori side information provided by the short-exposure image was utilized to improve the accuracy of kernel estimation, and then increased the effect of image restoration. For the kernel estimation, a priori filtering non-dimensional Gaussianity measure (BID-PFNGM) regularization term was raised, and the fidelity term was corrected using short-exposure image information, thus improving the kernel estimation accuracy. For the image restoration, a P norm-constrained relative gradient regularization term constraint model was put forward, and the restoration result realizing both image edge preservation and texture restoration effects was acquired through the further processing of the model results. The experimental results prove that, in comparison with other algorithms, the proposed algorithm has a better restoration effect.

## 1. Introduction

In the exposure process of an imaging detector, the relative motion between the image surface and object surface will lead to image motion and the degradation of image quality [1]. This motion problem is usually solved by two means: (1) the speed of moving the camera relative to the object surface is measured, and the image motion is compensated for by placing a fast reflection mirror in the light path [2]. By correcting the image motion directly through the principle of relative motion, this method is of favorable effect, but it will increase the cost and complexity of the camera hardware system. (2) The optical imaging mechanism is combined with the signal processing algorithm, and the image motion is compensated for using a deblurring algorithm [3]; this method is low-cost and easy to implement, but the kernel estimation accuracy of the image motion is greatly affected by the algorithm, which will impact the correction effect of the image motion.

A motion-deblurred image can be expressed as the projection of the convolution of the imaging target and the image motion kernel on the image surface. Therefore, a deblurring algorithm can be denoted as a deconvolutional process with an ill-posed problem [3]. If the shift image kernel is estimated only through the blurred image itself [3,4,5,6], the ill-posed problem will be serious in the equation solving process, with a large kernel estimation error, and it will not be easy to acquire good restoration results. To solve this problem, some scholars have proposed algorithms for multichannel deconvolution using two or more blurred images, such as in recent studies [7,8,9,10], and many scholars have proposed long- and short-exposure images, combining deblurring methods [11,12,13,14,15,16,17,18,19].

A deconvolutional algorithm was proposed in [11], which is based on a framework using short- and long-exposure images. The long-exposure images are motion-blurred, and short-exposure images are images obtained by controlling the exposure times until the relative motion is smaller than the scope of one image element. For short-exposure images, as the exposure time is short, the images are not blurred in the same way as motion-blurred images, but the contrast and signal-to-noise ratio (SNR) is poor. Therefore, the image motion of long-exposure blurred images can be corrected using the partial high-frequency information reserved by the short exposure, thus improving the estimation accuracy of the image motion kernel and mitigating the ill-posed problem of deconvolution. The method from [11] proved that the kernel estimation accuracy could be improved by including the short-exposure images in the estimation process as a priori side information. The regularization term design was simple in this kernel estimation algorithm; the kernel was acquired directly through the model derivation, but the ill-posed problem was not mitigated through more constraint methods, and the estimation result was not accurate enough [16]. In the image restoration part of this algorithm, an improved algorithm based on Richardson–Lucy (RL) was used, but the ringing effect problem still could not be well controlled. The image block restoration algorithm was also explored in [17,18], specific to the estimation problem of the space-varying kernel; the authors restored the images via the hierarchical Bayesian approach, but during the processing of the kernel estimation problem, the only constraint condition was that the energy sum of the kernel was 1. The model was directly solved through the constrained least-squares method, so the estimation accuracy of the image motion kernel was poor. Reference [19] proposed an improved RL algorithm to restore the images with short-exposure image information, to solve the deblurring problem of remote sensing images. In comparison with the restoration algorithm in [11], this algorithm improved the image restoration effect. However, the estimation method in the estimation part of the motion kernel was identical to that in [11], so, similarly, the estimation of the kernel was not accurate enough.

The problems existing in the present long- and short-exposure algorithms above, such as low kernel estimation accuracy and poor image restoration effect, were deeply studied in this research. The priori side information was mined using the correlation between two frames of images, and a new kernel estimation model and image restoration model were proposed, which relieved the ill-posed problem of the existing algorithms, improved the kernel estimation accuracy, and enhanced the image restoration effect. Future algorithms can significantly improve the imaging quality with the imaging results of the different camera lenses on deblurred scenarios involving devices, such as multi-lens smartphones, etc.

Section 2 introduces the main structure of the algorithm. Section 3 introduces the details of the modified algorithm proposed in this paper. Section 4 uses two groups of experiments to verify the effect of the algorithm.

## 2. Design Principle and Framework

### Basic Model of the Proposed Method

In general, imaging models for long- and short-exposure images can be expressed as below [11]:

(1) $$\begin{array}{l} Y_{L}^{Total}=H_{M}^{Total}⊛X^{Total}+N_{L}^{Total}\\ Y_{{}_{S}}^{Total}=X^{Total}+N_{S}^{Total} \end{array}$$

where ”⊛” denotes discrete convolution operator, $Y_{L}^{Total}$ is the long-exposure image, $Y_{{}_{S}}^{Total}$ is the short-exposure image, $H_{M}^{Total}$ is the blur kernel from the long-exposure image, $X^{Total}$ is an ideal real image, $N_{L}^{Total}$ and $N_{S}^{Total}$ denote the additive noises incurred in long-exposure and short-exposure imaging processes, respectively, both of which are zero-mean Gaussian noises by default. In the long- and short-exposure deconvolution framework, the ideal can be generally expressed as below [11]:

(2) $$X^{Total}=N_{d}^{Total}+\Delta X^{Total}$$

where $N_{d}^{Total}$ is the preprocessed image after the registration [20], straight variance balancing, and denoising [21] operations of the short-exposure image $Y_{{}_{S}}^{Total}$, aiming to enhance the information in the short-exposure image, which is consistently the same with the ideal image’s corresponding part, for example, the part of “Carton”(areas with large gradients in the image). $\Delta X^{Total}$ is the residual error of $X^{Total}$ and $N_{d}^{Total}$, and it is so small that it is usually ignored in the kernel estimation process. The deconvolutional algorithm model under the long- and short-exposure framework can be written into the following form:

(3) $$\begin{array}{l} H_{M}^{Total}=\underset{\forall H_{M}^{Total}\in {ℋ}_{M}}{\arg\min}{\left\|H_{}^{Total}⊛N_{d}^{Total}-Y_{L}^{Total}\right\|}_{2}^{2}\;\;\;\;\;\;\;\;\;\;(Estimate\;Kernel)\\ X_{opt}=\underset{\forall X^{Total}\in X}{\arg\min}{\left\|H^{Total}⊛X_{iter}^{Total}-Y_{L}^{Total}\right\|}_{2}^{2}\;\;\;\;\;\;\;\;\;\;\;\;\;(Estimate\;Original\;image) \end{array}$$

where ${\left\|\;\;\;\;\right\|}_{2}$ is the norm operator, and ${ℋ}_{m}$, X denote the pool of allowed $H_{M}^{Total}$ and $X_{iter}^{{}^{Total}}$. $X_{opt}$ is the preliminary restoration image. $H_{}^{Total}$ is the iter-kernel in the process, $X_{iter}^{{}^{Total}}$ is the iter-image in the process.

Under normal circumstances, this problem is an ill-posed problem, which cannot be directly solved, so it should be constrained by introducing the regularization method.

**Figure 1:**
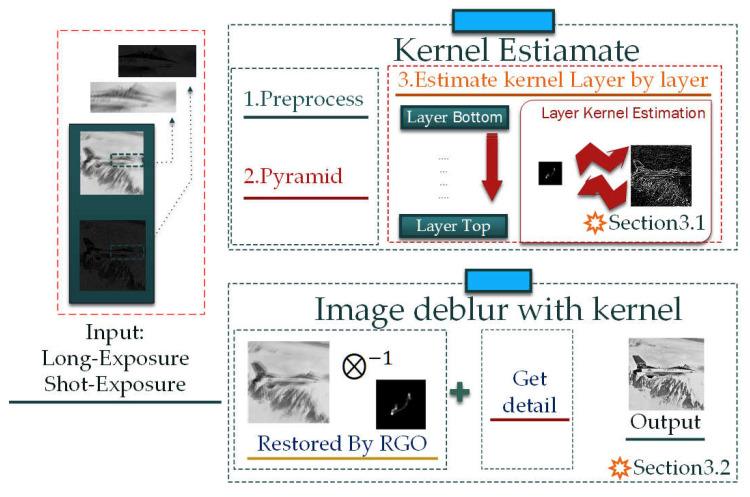
Structure flow chart.

As Figure 1 shows, the work of Equation (3) is divided into two parts: kernel estimation part and original image estimation part. Divide the problem to be solved by Equation (3) into two parts according to the steps shown in Figure 1.

Part 1: kernel estimation (introduced in Section 3.1).

The kernel estimation model in gradient field was obtained from Equation (3):

(4) $$H^{Total}=\underset{H^{Total}\in ℋ}{\arg\min}{\left\|h_{{}_{iter}}^{Total}⊛\;(\nabla N_{d}^{Total})-\nabla Y_{L}^{Total}\right\|}_{2}^{2}+\lambda \left\|\phi (h_{{}_{iter}}^{Total})\right\|$$

where λ is the regularization coefficient of kernel estimation, ℋ denote the pool of allowed $H_{}^{Total}$, $\phi (h_{{}_{iter}}^{Total})$ is the regularization term function for kernel estimation, $h_{{}_{iter}}^{Total}$ is the iter-kernel in the process (same as $H_{}^{Total}$). Because of the higher calculation efficiency, the kernel estimation model Equation (4) was solved by sparsity [22] in the image gradient domain. Therefore, the ‘’∇” means the function of the gradient operator.

The main work of Section 3.1 was to use short-exposure images to reconstruct the kernel estimation model Equation (4). By introducing the short-exposure images into the iterative image estimation process, with a new regularization term model proposed and the iterative optimization of data items, the accuracy of the kernel estimation were improved.

In detail, according to the solution framework in [3], first of all, the paper built an image pyramid by processing the blurred and short-exposure images through a stepwise downsampling method. Then, the kernel was estimated from the low-resolution image in the bottom layer of the pyramid. Next, the kernel estimation result of the low-resolution layer was used as the initial term of the kernel in the kernel estimation process of the upper high-resolution layer. Finally, by calculating layer-by-layer, the kernel estimation result of the original image was obtained. Since the estimation process is the same for every layer, Section 3.1 provides an example of the kernel estimation process of any layer to introduce the kernel estimation method in this paper.

Part 2: original image estimation by the part 1 result $H_{}^{Total}$ (introduced in Section 3.2)

The image deblur model in the spatial domain was obtained from Equation (3):

(5) $$X_{opt}^{}=\underset{X_{opt}^{}\in X_{opt}}{\mathrm{argmin}\;}{\left\|H_{M}^{Total}⊛X_{{}_{iter}}^{Total}-Y_{L}^{Total}\right\|}_{2}^{2}+\gamma_{X}\left\|R(X_{{}_{iter}}^{Total})\right\|$$

$\gamma_{X}$ is the regularization coefficient for an iter-restored image as a value, $X_{opt}$ is the pool of allowed $X_{opt}$, $X_{{}_{iter}}^{Total}$ is iter-image in the process, and $R(X_{{}_{iter}}^{Total})$ is the regularization term function for image restoration. In part 2, the paper used short-exposure images to design a new regularization term. The regularization term used the short-exposure image and the iter-restored image to jointly constrain the iterative solution process. This obtained a better restoration effect.

## 3. Main Works: Estimate Blur Kernel and Restore the Image Using Priori Side Information

As shown in the above section, in order to solve ill-conditioned problems, such as Equations (4) and (5), it is necessary to design appropriate models to highlight certain features of the image.

Aimed at the kernel estimation problem in Equation (4), traditional algorithms usually solve the equation with an easily designed regularization term. However, it is too difficult to obtain a good solution in this way, because of the nonconvex problem. Therefore, in this study, with the application of the expectation maximum, we obtained the estimation result of the kernel by the alternating layer-by-layer calculation of the layer iterative image and layer iterative kernel layer. Section 3.1 introduces the details of the method of kernel estimation in the selected layer with priori side information from the short-exposure image.

Aimed at the problem of Equation (5), Section 3.2 proposes a regularization term design method that uses short-exposure image information. The proposed regularization term, which retains information from the short-exposure image, is used to improve the restoration effect by retaining the advantage of the Lp-norm gradient operator, which is closer to the characteristics of natural image distribution.

### 3.1. Kernel Estimation in This Layer: Based on a Priori Filter of Short-Exposure Images to Build the Model

In this section, the estimation method for the kernel of example layer is introduced as an example for any layer of the image pyramid. As mentioned above, the main aim of the layer kernel estimation process is to apply the maximum expectation algorithm to estimate the layer iter-kernel and the layer iter-image iteratively. Therefore, for the target of improving the accuracy of the layer kernel estimation, we introduced the preprocessed layer short-exposure image as the correction template and used the JBF algorithm to calculate the result of correcting the current layer iter-image by the template. The result was the filtered iterative image for the current iteration step. As shown in Section 3.1.2 and Section 3.1.3, the a priori filtered iterative image could be applied to improve the regularization term design and optimize the fidelity term calculation results.

For the layer-kernel estimation problem, Equation (4) can be written in the following iterative form:

(6) $$H=\underset{\nabla X⇄h}{\mathrm{argmin}}{\left\|h_{iter}⊛X_{iter}-Y_{L}\right\|}_{2}^{2}+\lambda_{x}\left\|T(X_{iter})\right\|+\lambda_{h}\left\|F(h_{iter})\right\|$$

where *H* is the result of the kernel estimation in this layer, $Y_{L}$ is the deblurred image in this layer, and $\underset{a⇄h}{\mathrm{argmin}}$ represents the alternate iterative minimization of layer iterative image $X_{iter}$ and layer-iterative kernel $h_{iter}$ in this layer. The $\lambda_{{}_{X}}$ and $\lambda_{h}$ are regularization coefficients for iterative image estimation and kernel estimation in each layer. $T(X_{iter})$ and $F(h_{iter})$ are regularization terms for the layer iterative image estimation and layer-kernel estimation models, respectively.

**Figure 2:**
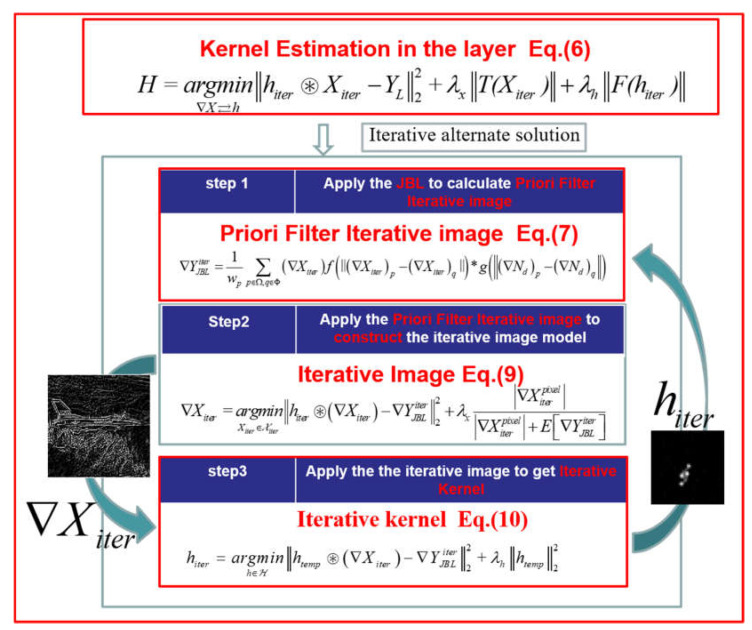
Flow chart of the kernel estimation model in the example layer.

As shown in Figure 2, the layer-kernel estimation process is mainly divided into three steps:

Step 1. Apply a joint bilateral filter (JBF) [23] algorithm to update the example layer’s priori filter image (Section 3.1.1).

Step 2. Apply the updated priori filter image to obtain a layer iterative image (Section 3.1.2).

Step 3. Apply the priori filter image to estimate the layer iterative kernel (Section 3.1.3).

The design requirement of the regularization term $T(X_{iter})$ in the layer iterative image solution process aims to improve the similarity between the gradient map of the layer iterative result and the gradient map ideal image. In Section 3.1.1, we propose a layer iterative image estimation model with short-exposure images, while optimizing the design of regularization and data items, and improving the estimation accuracy of iterative images.

The design requirements of the regularization term $F(h_{iter})$ in the layer iterative kernel solution process usually need to consider the feature of the kernel itself. In this paper, the common regularization term ${\left\|h_{iter}\right\|}_{2}^{2}$ is used to constrain the iterative kernel solution. The calculation process is simple, and the iterative result has a better overfitting control effect.

Therefore, by the improvements above, with the appropriate regularization term and continuously iterating alternately to convergence, the optimal solution of the iterative layer kernel H can be obtained.

#### 3.1.1. Calculate the Priori Filter Iterative Image

In this example layer, the gradient of the priori filter iterative image ”$\nabla Y_{JBL}^{iter}$” is obtained by the JBF of the iterative image $X_{iter}$ and the preprocessed image $N_{d}$:

(7) $$\begin{array}{l} \nabla Y_{JBL}^{iter}=\frac{1}{w_{p}}\sum_{p\in \Omega ,q\in \Phi }\left(\nabla X_{iter}\right)f\left(\left\|{\left(\nabla X_{iter}\right)}_{p}-{\left(\nabla X_{iter}\right)}_{q}\right\|\right)*g\left(\left\|{\left(\nabla N_{d}\right)}_{p}-{\left(\nabla N_{d}\right)}_{q}\right\|\right)\\ \;\;\;\;\;\;\;\;\;\;\;\;\;\;w_{p}=\sum_{p\in \Omega ,q\in \Phi }\left(\nabla X_{iter}\right)f\left(\left\|{\left(\nabla X_{iter}\right)}_{p}-{\left(\nabla X_{iter}\right)}_{q}\right\|\right)*g\left(\left\|{\left(\nabla N_{d}\right)}_{p}-{\left(\nabla N_{d}\right)}_{q}\right\|\right)\\ \;\;\;\;\;\;\;\;\;\;\;\;\;\;\;\;\;\;\;\;\;\;\;\;\;\;f(\cdot ),g(\cdot )=\exp([{\left(m_{x}-n_{x}\right)}^{2}+{\left(m_{y}-n_{y}\right)}^{2}]/2\delta^{2}) \end{array}$$

where $w_{p}$ is the normalization terms, *f*() and *g*(), are the spatial distance weight template function and the similarity weight template function, respectively, both of which are Gaussian functions in this algorithm with the default parameters δ. Φ is the range of the block filter area for example, [3,3], and *p* and *q* are the pixel coordinates; *p* is the center pixel of the block filtered area, *q* is the neighbor pixel of *p* in the block filter region, and Ω is the entire image. The values of *f*() and *g*() are both in the range [0, 1], so the result of the Hadamard product will be very small. Use of this result directly will result in severe distortion, so it is necessary to multiply it by a normalization factor, which is the inverse of the sum of all elements of the product matrix obtained by the Hadamard product from two matrices with the spatial distance weights and the similarity weights. ${\left(\nabla X_{iter}\right)}_{p}$ and ${\left(\nabla X_{iter}\right)}_{q}$ are the pixel gradient values of the iterative image. ${\left(\nabla N_{d}\right)}_{p}$ and ${\left(\nabla N_{d}\right)}_{q}$ are the pixel gradient values at the corresponding positions in $N_{d}$.

**Figure 3:**
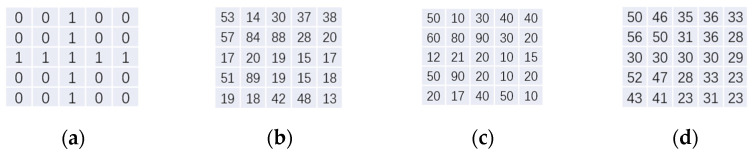
The matrix of examples: (**a**) template; (**b**) target; (**c**) bilateral filter; and (**d**) joint bilateral filter.

As the Figure 3 shown, assuming that there is a template matrix (a) and a target matrix (b), the same parameter settings are applied to obtain the bilateral filtering result (c) and the joint bilateral filter result (d). It is evident that the distribution of (d) is closer to template (a) than (c) and shows more details from (b) than template (a). This is determined by the principle of the joint bilateral algorithm. Therefore, it can be concluded that using $N_{d}$ images that are closer to the real distribution to participate in the calculation can make the distribution of the iterative results closer to the real distribution. The main reason why the $N_{d}$ image is not directly used in the calculation as a priori filter image is that the signal-to-noise ratio of the short-exposure image is low, and the detail loss in the smooth part of the image intensity transition is large. Therefore, using the results of bilateral filtering to participate in the iteration can have a good correction effect on both the texture and edge parts of the image.

For example:

#### 3.1.2. Iterative Image Estimation

The main concept in this section is that, with the priori filter non-dimensional Gaussianity measure regularization term, which was designed to constrain the layer iterative image estimation process, the iterative updating of the fidelity term was realized using the priori filtering term in the kernel estimation process. Compared with the common kernel estimation algorithms from the literature [11,15,18], this algorithm constrained the iteration process through the partial high-frequency information provided by short-exposure images, thus improving the kernel estimation accuracy.

The iterative process is divided into two steps:

Step 1: solve the iterative image model with BID-PFNGM regularization term and updated data item that introduces a priori filter image for reconstruction.

The BID-PFNGM is as below:

(8) $$T(X_{iter})=\frac{\left|\nabla X_{iter}^{pixel}\right|}{\left|\nabla X_{iter}^{pixel}\right|+E\left[\nabla Y_{JBL}^{iter}\right]}$$

where $E\left[\nabla Y_{JBL}^{iter}\right]$ means solving mathematical expectation, and the value is the mean of the gradients of all pixels of the priori filter image. As above, $\nabla Y_{JBL}^{iter}$ is the priori filter term, and the initial value is $N_{d}$. $\nabla X_{iter}^{pixel}$ is the pixel value of the selected location.

As the constraint term, BID-PFNGM can be used to judge the gradient information needing to be reserved or attenuated. If the gradient of pixel point is greater than the global mean of relative gradient, the pixel will be reserved, or otherwise abandoned. Through repeated updating of threshold and iterative Equation (9), only a small part of pixels was reserved at the end, which improved the kernel estimation accuracy while ensuring the sparsity.

The original NGM, which, from [24], was calculated iteratively in each iteration based on the previous result. The blur kernel used at the beginning was an artificial initial value, so the result of the initial iterative image deviated greatly from the final result. Although the final iterative result converged, if the mean value can be adjusted using the more reliable pixel gradient distribution provided by the short-exposure image in each iteration process and adding the priori distribution term and the fidelity term for calculation together (as shown in Equation (9)), the estimated effect of the final blur kernel is better. As described in 3.1.1, the addition of the joint bilateral filtering algorithm can make the value of the result of the corrected image have both the characteristics of the template image and the characteristics of the corrected image. If the template image has high reliability, then the corrected image is more credible than the uncorrected one.

Step 2: update the iterative image model.

Applying the priori filter image $Y_{JBL}$ and iterative image $X_{iter}$ to reconstruct the iterative image model $\nabla X_{iter}=\underset{X_{iter}}{\mathrm{argmin}}{\left\|h_{iter}⊛X_{iter}-Y_{iter}^{pixel}\right\|}_{2}^{2}+\lambda_{x}\left\|T(X_{iter})\right\|+\lambda_{x}\left\|T(X_{iter})\right\|$ from Equation (6) in gradient domain, it can be written as:

(9) $$\nabla X_{iter}=\underset{X_{iter}\in X_{iter}}{\mathrm{argmin}}{\left\|h_{iter}⊛\left(\nabla X_{iter}\right)-\nabla Y_{JBL}^{iter}\right\|}_{2}^{2}+\lambda_{x}\frac{\left|\nabla X_{iter}^{pixel}\right|}{\left|\nabla X_{iter}^{pixel}\right|+E\left[\nabla Y_{JBL}^{iter}\right]}$$

As in [24], $\lambda_{x}$ is a fixed value determined by experimental experience, $X_{iter}$ denote the pool of allowed $X_{iter}$. As mentioned before, the physical meaning of this regularization term is to keep a relatively large portion of the result compared to the mean of the priori filtered image. If the selection weight is too small, the final result will contain more noise information, and if the selection weight is too large, the resulting image information will be lost. Therefore, different images need different weight parameters according to the characteristics of the image (sharpness, texture, etc.), and the selection of this item is mainly determined by experimental experience.

To solve this problem, this paper used the iterative shrinkage-threshold (IST) algorithm, which has high calculation accuracy and a good restoration effect for non-uniform blur kernel [25]. After calculation, the iterative image solution is obtained: $X_{iter}$ [24].

#### 3.1.3. Iterative Kernel Estimation Algorithm

The iter-kernel $h_{iter}$ can be obtained by updating the kernel iteration model [26] using the priori filtering term $Y_{JBL}$ and iterative image $X_{iter}$:

(10) $$\begin{array}{c} h_{iter}=\underset{h\in ℋ}{\arg\min}{\left\|h_{temp}⊛\nabla X_{iter}-\nabla Y_{JBL}^{iter}\right\|}_{2}^{2}+\lambda_{h}{\left\|h_{temp}\right\|}_{2}^{2}\\ s.t.\sum h_{temp}^{pixel}=1\;and\;h_{temp}^{pixel}\geq 0 \end{array}$$

where $\lambda_{h}$ is a hyperparameter of the regularization term, ℋ denote the pool of allowed h, $h_{temp}^{}$ is the iter-temp result of $h_{iter}$, and the limitations are that the energy of the sum of all pixels from the kernel $\sum h_{temp}^{pixel}$ is 1, and the energy $h_{temp}^{pixel}$ of each point in the kernel is not less than 0. In this paper, we adopt a similar algorithm from [24] to obtain the iterative kernel by using the gradient projection method [26] to solve the equation. It is worth noting that the algorithm uses the Dirichlet function to approximate the true distribution of the iterative kernel [27]. From a statistical perspective, the process of solving the iterative kernel can be regarded as the process of solving the probability distribution of $h_{iter}$, when the probability of satisfying the conditional distribution $P(h|\nabla Y_{JBL})$ is the largest. However, it is very difficult to solve the model directly, so the probability of satisfying a certain Dirichlet approximate distribution $P(D_{Diriclet})$ is used to approximate $P(h|\nabla Y_{JBL})$, where $D_{Diriclet}$ represents the Dirichlet distribution to be determined. The relative entropy Kullback–Leibler (KL) distance is usually used to judge the similarity of two distributions. And as below in Table 1, the flow of the method is shown.

The proposed algorithm was compared with the algorithms in [11,24] (All results from [24,27] calculated by the code from URL: https://sites.google.com/site/fbdhsgp accessed on 6 December 2021. With GNU3.0 License. And the result of [11] calculated by the code from https://github.com/jtyuan/deblur accessed on 6 December 2021) in the aspect of kernel estimation, to verify its estimation accuracy. In the simulation process of the long-exposure image, the original image experienced blur processing using a kernel with the size of 6 pixels and in the direction of 45°. As for the short-exposure image, the exposure of the original image was reduced via PS software, the parameters were set as 30% of the original image, and Gaussian noise with a mean value of 0 and variance of 0.05 was added to simulate the low SNR characteristic of the short-exposure image.

**Figure 4:**
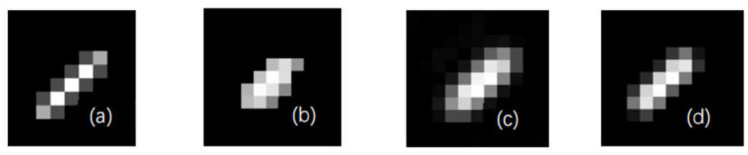

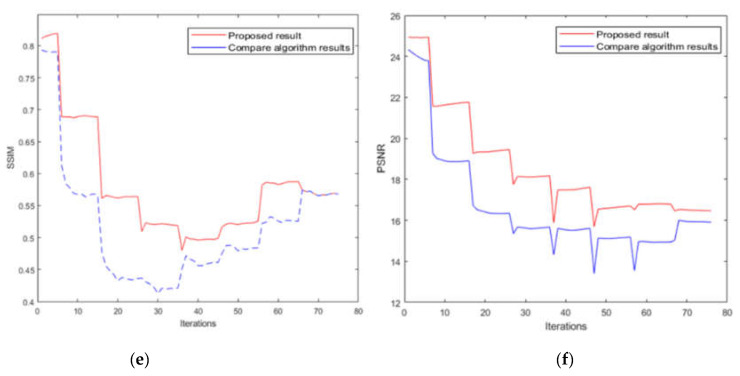
The kernel estimate result of (**a**) original kernel; (**b**) literature [11] algorithm result; (**c**) literature [24] algorithm result; (**d**) this paper; (**e**) normalized SSIM with [24]; (**f**)normalized PSNR with [24].

Figure 4 shows the kernel estimation results. It can be directly observed that the energy distribution of the kernel estimated by the proposed algorithm was more approximate to the original kernel. The results were evaluated using the quantitative index evaluation method as seen in Table 2. A comparative evaluation was conducted using two objective criteria: peak signal-to-noise ratio (PSNR, unit: dB) and kernel structural similarity (SSIM). Figure 4e,f show the PSNR and SSIM normalized comparison results of the blur kernel estimation results in the blur kernel estimation process. In the iterative process based on the image pyramid, due to down-sampling, the initial image was small and the value was high, and the overall trend was a downward trend. This was an inevitable result after resolution normalization. However, it did not affect the estimation effect of this algorithm in the entire iterative process, which was better than the results in [24]. It can be seen that the kernel estimation algorithm based on BID-PFNGM was better than the kernel estimation based on NGM. According to Table 2, the proposed algorithm showed better performance in both indexes, indicating that the estimation result of the proposed algorithm was superior to the algorithms in [11,24].

### 3.2. Image Deblur: Deconvolutional Restoration Based on Relative Gradient Operator

In the convolutional image restoration process, [17] used the global gradient function of the to-be-restored image as the constraint term. Affected by the staircase effect, the texture part in the image could not be well restored. References [11,18] used the RL algorithm to restore the image and obtain the original solution, but the RL algorithm had a poor image restoration effect, which also affected the final restoration effect.

To solve the above problems, in this paper we put forward an Lp-norm constraint function called the relative gradient operator (RGO) to construct the regularization. The regularization term constrained by the Lp-norm has a good edge preservation effect [28,29]. When the RGO was applied to the image, besides the global gradient information, the RGO contained the relative difference information between the iterative deblurred image and the short-exposure preprocessed image. Compared with the TV operator, which was defined as nonconvex in the image area, the texture information of the restoration result was clearer, and the effect was better. The following first gives the definition and properties of the RGO.

Any bounded Lipschitz region Ω in a two-dimensional image is defined as a domain interval [30], and the RGO can be written as follows:

(11) $$\begin{array}{l} CD\left(N_{d},X_{Riter}^{}\right)=\sqrt[{p}]{\int_{\Omega }{\left|N_{d}^{j}-X_{Riter}^{j-1}\right|}^{p}d\Omega }\\ \;\;\;\;\;\;\;\;\;\;\;\;\;\;\;\;\;=\sqrt[{p}]{{\left|\left(N_{d}^{j}-X_{Riter}^{j})+(X_{Riter}^{j}-X_{Riter}^{j-1}\right)\right|}^{p}d\Omega }\\ \;\;\;\;\;\;\;\;\;\;\;\;\;\;\;\;\;=\sqrt[{p}]{\int_{\Omega }{\left|\nabla X_{Riter}^{j}-X_{Riter}^{j}+N_{d}^{j}\right|}^{p}d\Omega } \end{array}$$

where a is the pixel in Ω, j is the location order of the pixel, and p is the power number in Section 3.2. Observing the above equation, it can be seen that the RGO must be integrable in the two-dimensional bounded Lipschitz bounded region (because this region is a two-dimensional real number field, and $N_{d}$ is a constant, the RGO must have an upper bound in this region, satisfying integrability) [31]; that is, it satisfies $\sqrt[{p}]{\int_{\Omega }{\left|\nabla X_{j}-X_{j}+N_{d}^{j}\right|}^{p}d\Omega }<\infty$, and the RGO has homogeneity.

Therefore, the RGO is a function defined on domain Ω belonging to LP space: $CD\in L^{p}(\Omega )$,and $L^{p}(\Omega )=\{u|\int_{\Omega }{\left|u(x)\right|}^{p}dx<\infty \}$, and *u* is any function that satisfies the condition:$\int_{\Omega }{\left|u(x)\right|}^{p}dx<\infty$ [31].

From the above equations, the relative gradient operator can be expressed as the sum of the summation operator and gradient operator; namely, the RGO contains the gradient information of the image itself and the difference information between the iterative deblur image and the priori image $N_{d}$. From the above equations, the relative gradient operator can be expressed as the sum of summation operator and gradient operator, namely, the RGO contains the gradient information of the image itself and difference information between iterative deblur image and priori image $N_{d}$.

So, it can be used to rewrite Equation (5) into the following form:

(12) $$X_{opt}=\underset{X_{Riter}\in X}{\arg\min}{\left\|H⊛X_{temp}-Y_{L}\right\|}_{2}^{2}+\gamma_{R}{\left\|CD\left(X_{Riter},N_{d}\right)\right\|}^{p}$$

where $\gamma_{R}$ is a hyperparameter of the regularization term, X denote the pool of allowed $X_{Riter}$, and $X_{temp}$ is the iterative temp value of $X_{opt}$. Equation (12) denotes the model of an objective function constrained by the relative gradient operator regularization term of the P-norm. It generally cannot be solved directly through the gradient descent-like method [32], so smooth approximation functions are usually used, such as the Huber function [33]:

(13) $$\mu_{\varepsilon }(v)=\{\begin{array}{ll} \frac{p}{2}\varepsilon^{p-2}v^{2}+\frac{2-p}{2}\varepsilon^{p}\;\;\;\;\;\;\;\;(if\;|v|<\varepsilon ) & \\ |v{|}^{p}\;\;\;\;\;\;\;\;\;\;\;\;\;\;\;\;\;\;\;\;\;\;\;\;\;\;\;\;\;\;\left(if\;|v|\geq \varepsilon \right) & \end{array}$$

where v is the independent variable and ε is the limited parameter, which determines the similarity between the Huber function and the original function.

Equation (12) is solved by approximately expressing it as a first-order continuously differentiable 2-norm function:

(14) $$X_{opt}=\underset{X_{Riter}\in X}{\mathrm{argmin}}{\left\|H⊛X_{temp}-Y_{L}\right\|}_{2}^{2}+\gamma \sum \mu \left(CD\left(X_{Riter}\right)\right)$$

As $N_{d}$ is a constant, for any p>0, Equation (12) is first-order continuous differentiable. When ε is sufficiently small, the solutions of Equations (12) and (14) are approximately equivalent. $CD\left(X_{Riter},N_{d}\right)$ can be written into a function $CD(X_{Riter})$ about $X_{Riter}$. When Equation (12) reaches the minimum value, the following can be obtained through the derivation:


(15) $${\left\|H\right\|}^{2}⊛X_{temp}-\|H\|⊛Y_{L}+\gamma_{R}M{\left\|CD(X_{Riter})\right\|}_{2}^{2}=0$$

M is a diagonal matrix similar to $diag(min(p\varepsilon^{p-2},p|CD(X_{Riter}){|}^{p-2}))$.

With the introduction of auxiliary function T(a) [34] and based on the idea of majorization minimization (MM) [35], this problem can be solved by the alternating direction method of multipliers (ADMM) [36], so as to obtain the initial solution X.

After the initial solution was solved, the high-frequency detailed information of the image was extracted to enhance its texture presentation. The extraction of high-frequency detail information was solved by a similar method to that in [18].

First, the residual error image and residual error image $\Delta Y_{L}=Y_{L}-H⊛N_{d}$ were used as the inputs. Then the residual error solution $X_{RRL}$ and gain control solution $X_{gcl}$ were acquired through the residual Richardson–Lucy (RRL) [37] and gain control Richardson–Lucy (GCRL) [38] algorithms, respectively. Secondly, the united bilateral filtering of $X_{RRL}$ and the initial solution $X_{opt}$ were conducted to acquire the smooth image $\bar{X}$, followed by the operation of $X_{detail}=X_{g}-\bar{X}$ to acquire the detailed image solution $X_{detail}$. In the end, the initial solution and detailed solution were integrated ($X=X_{opt}+X_{detail}$) to obtain the final restored image X. The flow of the whole algorithm in Section 3.2 was shown in Table 3.

**Figure 5:**
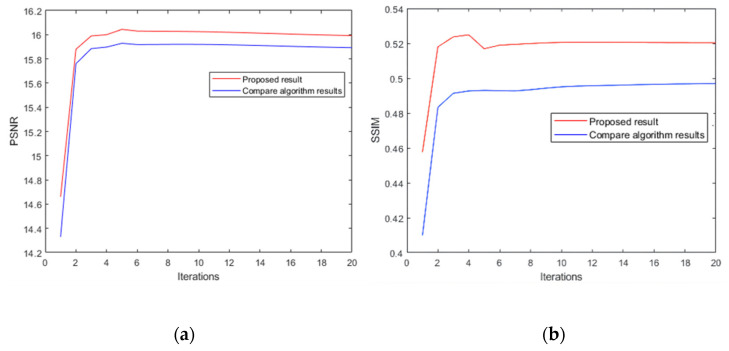
Comparison of iteration: (**a**) PSNR result of difference; (**b**) SSIM result.

As shown in Table 4 and Figure 5, in the case of using the same kernel, we used the PSNR and SSIM indicators to compare the results of the restoration effect of the algorithm in this paper and the algorithm in [27]. Table 4 gives a comparison of the parameters of the final restoration result, and Figure 4 shows a comparison of the relevant parameters of the iterative restoration image in each iteration. The top row of Figure 4 shows the difference in the PSNR of the restored iterative image, and the bottom row of Figure 4 shows the comparison of the SSIM of the restored iterative image. It can be seen that the proposed algorithm works well. Both of the results were better than those in [27].

## 4. Results and Discussion

In this paper, three groups of experiments were used to examine the performance of our algorithm. The first group comprised simulation experiments, which aimed to examine the restoration effect of this algorithm by measuring the quality evaluation index with reference images. The second group of experiments, named the live shot group, were conducted to verify the practicability of the algorithm by examining its restoring effect on live-photography images. The live shot group consisted of experiment 1 and experiment 2, where the algorithms in [11,17] constituted the first control group in experiment 1—group 2, and those in [11,27] formed experiment 2—group 2. In the third group, composed of the modulation transfer function (MTF) measurement experiments [39], the curve of the image was measured by restoring the simulation image.

### 4.1. Group 1: Simulation Experiment

Experiment 1: the first group of the simulation experiment used remote sensing images with an image size of 512 × 512 pixels, and the long-exposure images were manually blurred. The short-exposure image used PS software to reduce the exposure of the original image, the exposure was set to 30% of the original image, and Gaussian noise with a mean value of 0 and a variance of 0.05 was added to simulate the low signal-to-noise ratio of the short-exposure image.

**Figure 6:**
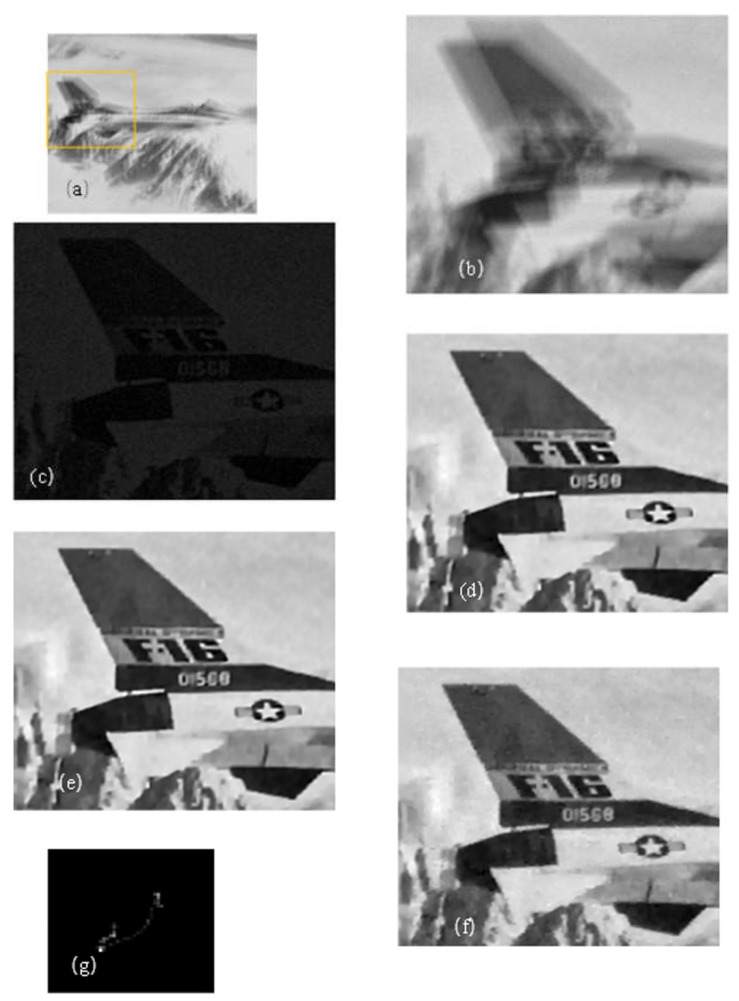
Remote image restoration: (**a**) blurred image; (**b**) local blurred image; (**c**) local Scheme 11. algorithm restored image; (**e**) local [27] algorithm restored image; (**f**) local restoration result of this algorithm; (**g**) the kernel estimated by this paper.

The experimental results are shown in Figure 6. It can be seen by directly observing the restoration results that the methods in [11,27] were not accurate enough in the kernel estimation. However, the proposed algorithm had better performance in both edge preservation and texture restoration. This group of experiments comprised simulation experiments, and there was an original image, so the reference image quality index was used to measure the quality of the restored image. This group of experiments used SSIM and PSNR as indicators for measurement. As shown in Table 5, this algorithm performed well in both metrics.

Experiment 2: the second group of the simulation experiment used Image Library, and the long-exposure images were manually blurred. The short-exposure image used PS software to reduce the exposure of the original image, the exposure was set to 30% of the original image, and Gaussian noise with a mean value of 0 and a variance of 0.05 was added to simulate the low signal-to-noise ratio of the short-exposure image. The main purpose of this experiment is to verify the generalizability of this algorithm, so this algorithm used eight kernels to blur all four images of Levin’s image library for blur simulation. The test metrics were selected as PSNR and SSIM to measure the overall performance of each algorithm in each image. The specific calculation method used the summation method, and the PSNR and SSIM values of the recovered images of the eight kernels for each image were summed up individually, and the total value was used as the final evaluation result among them, the PSNR parameters processed by histogram matching with the original image.

**Figure 7:**
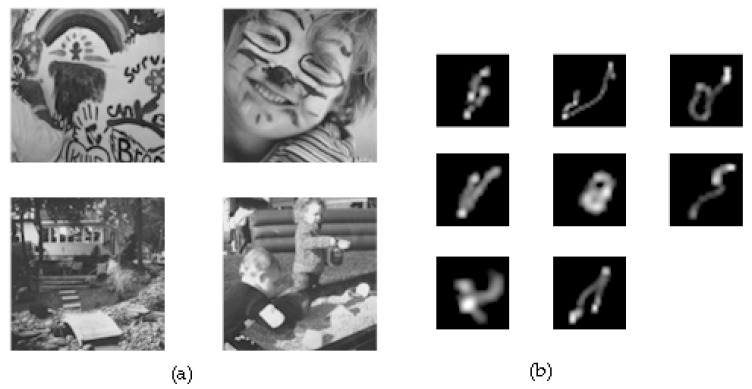
The Levin library; (**a**) the original images from Levin library; (**b**) kernels.

Figure 7 shows (a) the original state images of the images and (b) the self-designed simulation blur kernel. As shown in Table 6 and Table 7, the algorithm in this paper performs well overall in both PSNR and SSIM metrics of the images; combined with the recovery effect shown in Figure 6, it can be believed that the algorithm in this paper can outperform the comparison algorithm in most scene fields in both visual intuitive perception and objective metric measurements, on most occasions.

### 4.2. Group 2: Live Shot Group, Experiment 1

This group of experiments comprised real-shot experiments, so the subjective evaluation method was used to evaluate the restoration quality of the image. However, based on some existing image quality assessment (IQA) indicators, such as natural image quality evaluator(NIQE, the smaller the value, the smaller the gap with the natural image, the better the image quality) [40], average gradient (the larger the value, the clearer the image texture), cumulative probability of blur detection (CPBD, the larger the number, the sharper the image) [41], these three indicators could also evaluate the image restoration quality from another perspective.

The live shot data in [17] were used in the first control group, and the results were compared with the processing results in [11,17]. The live shot image was from [17], page 4, with a size of 498 × 498 pixels, and the copyright belongs to the original author, Tallon.

**Figure 8:**
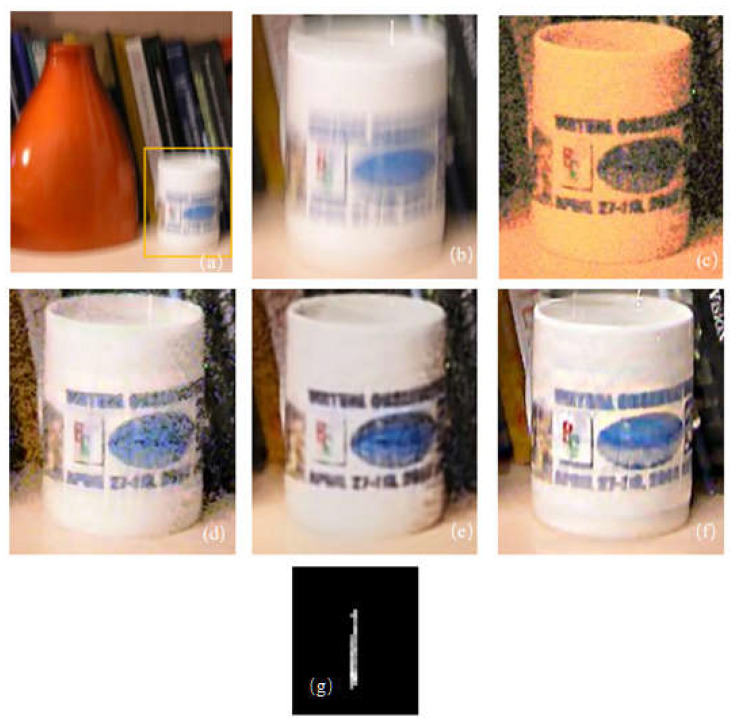
Real image restored experiment; (**a**) blurred image; (**b**) blurred image part; (**c**) Scheme 11. (**e**) algorithm restored image in [17]; (**f**) this paper; (**g**) kernel by proposed.

The results are shown in Figure 8 and Table 8. It can be seen by directly observing the restoration results that the methods in [11,17] were not accurate enough in the kernel estimation; the yellow spine on the left in the figure is quite fuzzy, as is the pattern edge on the cup. Moreover, the characters in the upper right corner (almost) cannot be identified. However, the proposed algorithm had better performance in both edge preservation and texture restoration. Through subjective observation, combined with measurement of the IQA index, it can be seen that the recovery performance of this algorithm was better. The restored image texture is clearer, the larger gradient part is sharper, and the visual effect is better.

### 4.3. Group 2: Live Shot Group Experiment 2

The second experimental picture was taken by an industrial camera (The Imaging Source DFK290). In order to test the result of the proposed algorithm restored from a natural scene in bad imaging conditions, we selected the exposure time and ISO of the long-exposure image as 1/47 s and 100, respectively. The length and width of a single CMOS pixel were 3 μm, the focal length was 50 mm, and the object distance was 5 m. The image resolution was 1920 × 1080, and the area with a resolution of 400 × 360 was intercepted for the restoration. The exposure time and ISO of the short-exposure image were 1/1600 s and 400, respectively. After calculating the camera and lens parameters, the upper speed limit, at which the relative motion would not be generated within the shutter time, was determined to be about 1.7 m/s; in other words, no motion-induced fuzziness would be generated if the relative motion speed were lower than this speed limit. Through the test, the relative motion speed in the experiment was much higher than 1.7 m/s, so the shutter time selected was suitable and conformed to the short-exposure imaging requirement. The relative motion-triggered fuzziness was large in this experiment, and the algorithm in [11] followed different principles from the proposed restoration algorithm, so an obvious relative position difference existed in the restoration result. 

**Figure 9:**
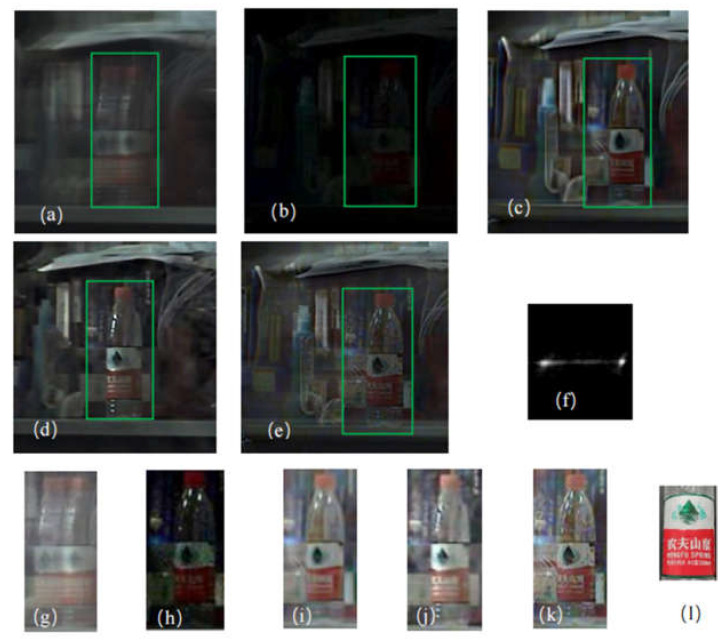
Real image results, the important part of the image comprises the Chinese characters as shown in (**l**): (**a**) blurred image; (**b**) short exposure image; (**c**) algorithm restored image in [27]; (**d**) algorithm restored image in [11]; (**e**) the restoration result of this algorithm; (**f**) result of kernel; (**g**) blurred image; (**h**) short exposure image; (**i**) algorithm restored image in [27]; (**j**) algorithm restored image in [11]; (**k**) algorithm proposed in this paper; (**l**) details of the Chinese characters.

The experimental results by the IQA are seen in Table 9:

The experimental results are seen in Figure 9. It can be directly observed that the image restored using the algorithm in [11] generated too smooth an effect, with great loss of texture details. The restoration effect of [27] was improved somewhat, but the characters on the bottle still could not be identified. Through subjective observation combined with the measurement of the IQA index, it can be seen that the recovery performance of this algorithm was better. The image restored using the proposed algorithm presented clearer edges than the results obtained through the previous two algorithms; for example, the character profile on the shot object was clearer, there were more texture details, and the image was more real and natural without obvious ringing effects; moreover, it was richer in colors.

### 4.4. Group 3: MTF Measurement Experiment

The sharpness of the image restored by the proposed algorithm was evaluated through the MTF in this group of experiments. The MTF parameters were measured through the method conforming to ISO12233 [39]. The blade edge image (420 × 650) was selected as the original image, and the motion-induced fuzziness of a long-exposure image was simulated using a 45° kernel with a pixel size of 6. The short-exposure image was simulated by reducing the exposure of the original image via PS software. The input–output ratio of the brightness parameter curve was adjusted to 36:89, the exposure was reduced to 30% of the original image, and Gaussian noise with a mean value of 0 and variance of 0.05 was added to simulate the low SNR characteristic of a short-exposure image. The measurement algorithm implemented the calculation by selecting the program (SFR_1.41) compiled by MITRE Corporation in 2006.

**Figure 10:**
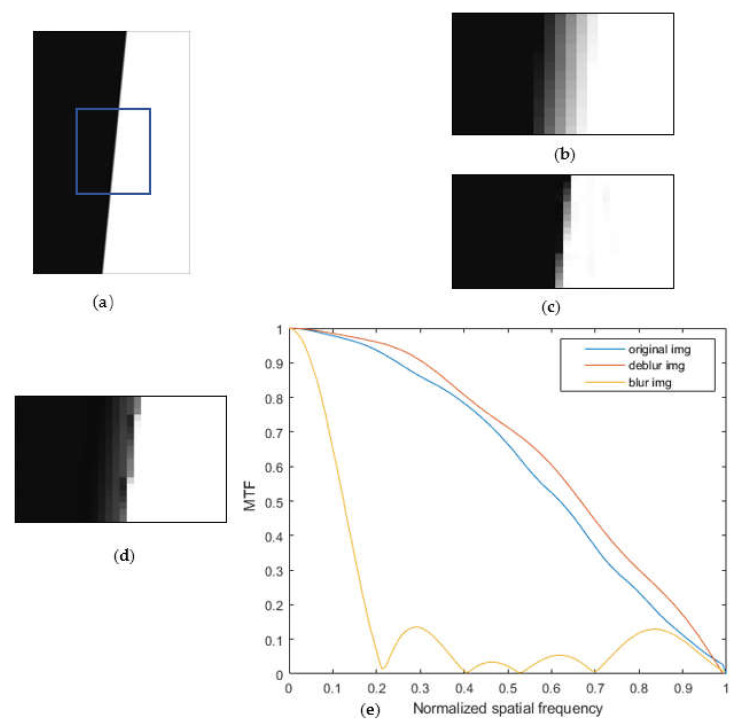
Edge image restoration results: (**a**) blurred image; (**b**) enlarged blurred image; (**c**) original image; (**d**) enlarged results; and (**e**) MTF comparison.

The experimental results are displayed in Figure 10. The main design goal of this experiment was to evaluate the restoration effect of this algorithm by measuring the MTF index of the deblur image. The test program was used to determine the MTF-related value by calculating the smoothness of the transition part in the longitudinal direction of the edge area. By observing the restored image (d) and the original image (c), the transition of the deblur image in the longitudinal direction is more obvious, and the original image is smoother and fuzzier. Therefore, in the MTF comparison chart, the MTF of the image deblurred by this algorithm was better than the MTF of the original image. The results show that the MTF of the proposed algorithm improved the edge sharpness effectively, with an excellent restoration effect, reaching the expectation of the algorithm design.

## 5. Conclusions

The advantages and disadvantages of single-image blind deconvolution algorithms and multi-image deconvolution algorithms were firstly analyzed, and then the merits of the long- and short-exposure deblur algorithm and the blind deblur algorithm were integrated to propose a deconvolutional deblur solution with practical value. Through the united kernel estimation of a short-exposure image and a long-exposure image, the priori side information provided by the short-exposure image was fully utilized, and the morphology of kernel was accurately estimated in the estimation part; in the image restoration part, a new regularization term was designed using the short-exposure image, the image details were enriched by introducing the detailed image information, and the image edges were kept sharp, thus effectively improving the final image restoration effect. The final experimental results also provide that the proposed algorithm basically reaches the design expectation, and the restoration effect of the existing algorithm is apparently enhanced.

## Figures and Tables

**Table 1:** Iterative estimation algorithm flow of kernel final.

The Steps of the Flow
Input: $N_{d}$, Y, $\lambda_{x}$ and $\lambda_{h}$
1. Conduct image d-sampling and establish an image pyramid consisting of *n* layers.
2. Estimate the convolution kernel at the current layer.
3. Update the fidelity term and regularization term through the united filtering algorithm.
4. Estimate the iterative image.
5. Estimate the convolution kernel.
6. Interpolate both kernel and image and extend to the next layer and repeat Step 2.
7. End the estimation and return to kernel $H_{opt}$.

**Table 2:** Kernel estimation quality evaluation table.

Test Image	Evaluation Criterion	The Algorithm in the Literature [11]	The Algorithm in the Literature [24]	Proposed Algorithm
Convolutional kernel restoration result	SSIM	0.6212	0.7835	0.8014
	PSNR	15.6289	16.3289	17.6426

**Table 3:** Deconvolutional algorithm flow based on long- and short-exposure.

Deconvolutional Algorithm Flow Based on Long- and Short-Exposure
Input: $Y_{L}$, $N_{d}$ and H
1. Calculate the initial value of regularization term
2. Iteratively solve Equation (15) to acquire the initial solution $X_{opt}$
3. Calculate the result of the RRL algorithm “$X_{RRL}$”
4. Calculate the result of GCRL algorithm “$X_{gcl}$”
5. Calculate the image details “$X_{detail}$” and acquire the restoration result “X”

**Table 4:** Final result of RGO deblur and [27].

Test Image	Evaluation Criterion	Algorithm in the Literature [27]	Proposed Algorithm
Convolutional kernel Restoration result	PSNR	26.2330	26.5231
	SSIM	0.7519	0.7707

**Table 5:** Final result of simulation experiment.

Test Image	Evaluation Criterion	Algorithm in the Literature [11]	Algorithm in the Literature [27]	Proposed Algorithm
Jet	SSIM	0.9768	0.9892	0.9992
	PSNR	25.4328	25.4251	26.8518

**Table 6:** Final results of total PSNR.

Test Image	Algorithm in the Literature [11]	Algorithm in the Literature [27]	Proposed Algorithm
Image 1	144.4002	145.6224	159.2353
Image 2	144.8377	152.0045	157.8592
Image 3	140.7298	147.0867	153.9975
Image 4	117.0562	143.5941	144.8717

**Table 7:** Final results of total PSNR.

Test Image	Algorithm in the Literature [11]	Algorithm in the Literature [27]	Proposed Algorithm
Image 1	6.3098	6.7155	6.7284
Image 2	6.6004	6.6171	6.6784
Image 3	6.3357	6.7661	6.8571
Image 4	4.3364	6.3227	6.5260

**Table 8:** Final results of the no. 1 experiment.

Test Image	Evaluation Criterion	Algorithm in the Literature [11]	Algorithm in the Literature [17]	Proposed Algorithm
Bottle	NIQE	5.3634	6.6016	4.4486
	Average Gradient	7.5376	5.0055	10.9265
	CPBD	0.4962	0.2182	0.5753

**Table 9:** Final results of the no. 2 experiment.

Test Image	Evaluation Criterion	Algorithm in the Literature [11]	Algorithm in the Literature [27]	Proposed Algorithm
Bottle	NIQE	4.3606	4.7263	4.1259
	Average Gradient	3.6899	3.6745	4.2400
	CPBDM	0.4962	0.2182	0.5753

## Data Availability

Not applicable.

## References

[1] Gonzalez R.C., Woods R.E. (2018). Digital Image Processing.

[2] Catanzaro B.E., Thomas J.A., Cohen E.J. (2001). Comparison of full-aperture interferometry to subaperture stitched interferometry for a large-diameter fast mirror. Optomech. Des. Eng..

[3] Freeman W.T., Fergus R.D., Singh B., Hertzmann A.P., Roweis S.T. (2019). Removing Camera Shake from a Single Photograph Using Statistics of a Natural Image. U.S. Patent.

[4] Bishop T.E., Babacan S.D., Amizic B., Katsaggelos A.K., Chan T., Molina R. (2007). Blind Image Deconvolution: Problem regaluration and existing approaches. Blind Image Deconvol. Theor. Appl..

[5] Qi S., Jia J., Agarwala A. (2008). High-quality motion deblurring from a single image. Acm Trans. Graph..

[6] Pan Z., Tan Z., Lv Q.B. (2017). Improved joint deblurring algorithm in Fourier domain and wavelet domain. Acta Photonica Sin..

[7] Yang L., Ji H. A Variational EM Framework With Adaptive Edge Selection for Blind Motion Deblurring. Proceedings of the IEEE/CVF Conference on Computer Vision and Pattern Recognition (CVPR).

[8] Li W., Zhang J., Dai Q. Exploring aligned complementary image pair for blind motion deblurring. Proceedings of the 2011 IEEE Conference on Computer Vision and Pattern Recognition (CVPR).

[9] Li Z., Deshpande A., Xin C. Denoising vs. deblurring: HDR imaging techniques using moving cameras. Proceedings of the 2010 IEEE Computer Society Conference on Computer Vision and Pattern Recognition.

[10] Roubek F., Flusser J. (2005). Resolution enhancement via probabilistic deconvolution of multiple degraded images. Pattern Recognit. Lett..

[11] Yuan L., Sun J., Quan L., Shum H.Y. (2007). Image deblurring with blurred noisy image pairs. ACM SIGGRAPH 2007 Papers, Proceedings of the SIGGRAPH07: Special Interest Group on Computer Graphics and Interactive Techniques Conference, San Diego, CA, USA, 5–9 August 2007.

[12] Bentum M.J., Arendse R.G., Slump C.H., Mistretta C.A., Peppler W.W., Zink F.E. Design and realization of high speed single exposure dual energy image processing. Proceedings of the Fifth Annual IEEE Symposium on Computer-Based Medical Systems.

[13] Gao Z., Yao S., Yang C., Xu J. (2015). A Dynamic Range Extension Technique for CMOS Image Sensors With In-Pixel Dual Exposure Synthesis. IEEE Sens. J..

[14] Vengsarkar A.M., Zhong Q., Inniss D., Reed W.A., Lemaire P.J., Kosinski S.G. (1994). Birefringence reduction in side-written photoinduced fiber devices by a dual-exposure method. Opt. Lett..

[15] Tallón M., Javier M.A., Babacan S.D., Katsaggelos A.K. (2013). Full Length Article Space-variant blur deconvolution and denoising in the dual exposure problem. Inf. Fusion.

[16] Li X.C., Li H.K., Song B. (2014). Application of energy functional regularization model in image restoration. J. Image Graph..

[17] Tallón M., Mateos J., Babacan S.D., Molina R., Katsaggelos A.K. Space-variant kernel deconvolution for dual exposure problem. Proceedings of the 19th European Signal Processing Conference.

[18] Zhang G.M., Gao S., Yin Z.S. (2017). Motion blur restoration method of remote sensing image based on fuzzy image and noise image. Electron. Des. Eng..

[19] Cui G., Hua W., Zhao J., Gong X., Zhu L. (2018). A motion deblurring method with long/short exposure image pairs. International Conference on Optical Instruments and Technology 2017: Optoelectronic Imaging/Spectroscopy and Signal Processing Technology, Proceedings of the International Conference on Optical Instruments and Technology 2017, Beijing, China, 28–30 October 2017.

[20] Leutenegger S., Chli M., Siegwart R.Y. BRISK: Binary Robust invariant scalable keypoints. Proceedings of the International Conference on Computer Vision.

[21] Djurovi I. (2016). BM3D filter in salt-and-pepper noise removal. EURASIP J. Image Video Process..

[22] Osher S., Burger M., Goldfarb D., Xu J., Yin W. (2005). An iterative regularization method for total variation-based image restoration. Siam J. Multiscale Model. Simul..

[23] Xiao C., Gan J. (2012). Fast image dehazing using guided joint bilateral filter. Vis. Comput..

[24] Xu Z., Fugen Z., Bei X.Z. Blind deconvolution using a nondimensional Gaussianity measure. Proceedings of the 2013 IEEE International Conference on Image Processing.

[25] Beck A., Teboulle M. (2009). A Fast Iterative Shrinkage-Thresholding Algorithm for Linear Inverse Problems. Siam J. Imaging Hences.

[26] Nocedal J., Wright S. (1999). Numerical Optimization. Springer Sci..

[27] Zhou X., Mateos J., Zhou F., Molina R., Katsaggelos A.K. (2015). Variational Dirichlet Blur Kernel Estimation. IEEE Trans. Image Process..

[28] Krishnan D., Fergus R. (2009). Fast image deconvolution using Hyper-Laplacian prioris. Adv. Neural Inf. Process. Syst..

[29] Levin A., Fergus R., Durand F., Freeman W.T. (2007). Image and Depth from a Conventional Camera with a Coded Aperture. ACM Trans. Graph. (TOG).

[30] Mumford D., Gidas B. (2001). Stochastic Models for Generic Images. Q. Appl. Math..

[31] Chen F.C., Shen J.H., Chen W.B. (2011). Image Processing and Analysis: Variational, PDE, Wavelet and Stochastic Methods.

[32] Kloft M., Brefeld U., Sonnenburg S., Zien A. (2011). *Lp*-Norm Multiple Kernel Learning. J. Mach. Learn. Res..

[33] Babacan S.D., Molina R., Do M.N., Katsaggelos A.K. (2012). Bayesian Blind Deconvolution with General Sparse Image Prioris. European Conference on Computer Vision.

[34] Giusti E., Williams G.H. (1984). Minimal Surfaces and Functions of Bounded Variation.

[35] James G., Witten D., Hastie T., Tibshirani R. (2013). An Introduction to Statistical Learning: With Applications in R (Springer Texts in Statistics).

[36] Boyd S., Parikh N., Chu E., Peleato B., Eckstein J. (2010). Distributed Optimization and Statistical Learning via the Alternating Direction Method of Multipliers. Found. Trends Mach. Learn..

[37] Faisal M., Lanterman A.D., Snyder D.L., White R.L. (1995). Implementation of a modified Richardson-Lucy method for image restoration on a massively parallel computer to compensate for space-variant point spread of a charge-coupled-device camera. J. Opt. Soc. Am. A.

[38] Cui G., Zhao J., Gao X., Feng H., Chen Y. (2017). High quality image-pair-based deblurring method using edge mask and improved residual deconvolution. Opt. Rev..

[39] (2017). Photography Electronic Still Picture Imaging Resolution and Spatial Frequency Responses.

[40] Mittal A., Soundararajan R., Bovik A.C. (2013). Making a “Completely Blind” Image Quality Analyzer. IEEE Signal Processing Lett..

[41] Narvekar N.D., Karam L.J. (2011). A No-Reference Image Blur Metric Based on the Cumulative Probability of Blur Detection (CPBD). IEEE Trans. Image Process..

